# Home gardens’ agrobiodiversity and owners’ knowledge of their ecological, economic and socio-cultural multifunctionality: a case study in the lowlands of Tabasco, México

**DOI:** 10.1186/s13002-020-00392-2

**Published:** 2020-07-20

**Authors:** Teresita Avilez-López, Hans van der Wal, Elda Miriam Aldasoro-Maya, Ulises Rodríguez-Robles

**Affiliations:** 1 Recursos Naturales y Desarrollo Rural, El Colegio de la Frontera Sur, Carretera a Reforma Km. 15.5, Ranchería Guineo 2da. Sección, 86280 Villahermosa, Tabasco México; 2 El Colegio de la Frontera Sur, Carretera a Reforma Km. 15.5, Ranchería Guineo 2da. Sección, 86280 Villahermosa, Tabasco México; 3 Departamento de Ecología y Recursos Naturales, Centro Universitario de la Costa Sur, Universidad de Guadalaja, Av Independencia Nacional 151, Centro, 48900 Autlán de Navarro, Jalisco, Mexico

**Keywords:** Biocultural diversity, Species richness, Shannon diversity index, Knowledge dialogue, Multicriteria analysis

## Abstract

**Background:**

Home gardens (HGs) are hotspots of in situ agrobiodiversity conservation. We conducted a case study in Tabasco, México, on HG owners’ knowledge of HG ecological, economical and socio-cultural multifunctionality and how it relates to agrobiodiversity as measured by species richness and diversity. The term multifunctionality knowledge refers to owners’ knowledge on how HGs contribute to ecological processes, family economy, as well as human relations and local culture. We hypothesized a positive correlation between owners’ multifunctionality knowledge and their HGs’ agrobiodiversity.

**Methods:**

We inventoried all perennial species in 20 HGs, determined observed species richness, calculated Shannon diversity indexes and analysed species composition using non-metric multidimensional scaling (NMDS). Based on literature, semi-structured interviews and a dialogue of knowledge with HG owners, we catalogued the locally recognized functions in the ecological, economic and socio-cultural dimensions. We determined the score of knowledge on each function in the three dimensions on explicit scales based on the interviews and observed management. We determined Spearman *rs* correlations of HGs’ observed species richness, Shannon diversity index (H) and of HGs’ scores on NMDS-axis and multifunctionality knowledge scores. We dialogued on the results and implications for agrobiodiversity conservation at workshops of HG owners, researchers and local organizations.

**Results:**

HG agrobiodiversity and owners’ multifunctionality knowledge in the study area showed large variation. Average richness was 59.6 perennial species, varying from 21 to 107 species, and total observed richness was 280 species. A total of 38 functions was distinguished, with 14, 12 and 12 functions in the ecological, economic and socio-cultural dimensions. Total multifunctionality knowledge scores varied from 64.1 to 106.6, with an average of 87.2. Socio-cultural functionality knowledge scores were the highest, followed by scores in the ecological and economic dimensions. Species richness and Shannon H were significantly correlated with ecological functionality knowledge (*rs* = 0.68 and *P* < 0.001 in both cases), and species richness was also correlated with economic functionality knowledge (*rs* = 0.47, *P* = 0.03). Species composition scores on the first and second axes of NMDS was significantly correlated with knowledge of ecological multifunctionality, with *rs* = 0.49 resp-0.49 and *P* = 0.03 in both cases. Other functionality knowledge scores showed no correlation with NMDS scores. Dialogue in workshops confirmed the interwovenness of multifunctionality knowledge and agrobiodiversity.

**Conclusion:**

The rich agrobiodiversity of home gardens cherished by rural families in Tabasco relates with the knowledge about HG functionality in the ecological and economic dimensions. Also, species composition relates with ecological functionality knowledge. The socio-cultural functionality knowledge, which includes many elements beyond the individual HG, is not correlated with agrobiodiversity, but had the highest scores. Our results show that multifunctionality knowledge provides many opportunities for the participative conception and planning of policies and actions necessary to conserve agrobiodiversity.

## Background

Tropical home gardens (HGs) are socio-ecological systems that maintain a high diversity of cultivated, enhanced and tolerated species, usually denominated agrobiodiversity, and contribute to in situ conservation of plant genetic resources and ongoing processes of domestication [1–7]. HGs are part of cultural landscapes, i.e. areas that give meaning and identity to their habitants and are shaped by culture through its inextricable relation with the managed and unmanaged environment [8–10]. Agrobiodiversity goes through selection and management processes by the HG owners as they fingerprint their culture on home gardens in daily practice [11–15] and adapts to the varied microclimate and soil conditions in the complex, forest-like agroecosyestem [4, 16, 17], taking part in manifold interactions at genetic, species, ecosystem and landscape scales [11, 18–21].

HGs’ agrobiodiversity depends on the continuous management, experimentation, cultivation, organization, knowledge transmission and motivations of their owners [3, 22, 23]. Their species composition and vegetation structure respond to ecological, economical and socio-cultural functions that local people aim at through design and management at different scales [11, 23–26]. In this regard, our use of the term “functions” refers to those operating in socio-ecological systems at scales from sections of agroecosystems to the landscape [19, 27–29]. Functions derive from the ecological, economic and social system dimensions, as well as their interactions, such as learning about agrobiodiversity [23, 26, 30, 31]. Ecological functions refer to the functions that also occur in natural ecosystems, such as nutrient cycling, enhancing rainwater infiltration in soils, generating distinct micro-climates and providing habitats to species. Economic functions refer to generating products and services for human consumption, favouring family economy through income and savings. Socio-cultural functions refer to the enhancement of social relations and aesthetic, learning, spiritual and emotional functions, among others [2, 26, 29].

**Fig. 1:**
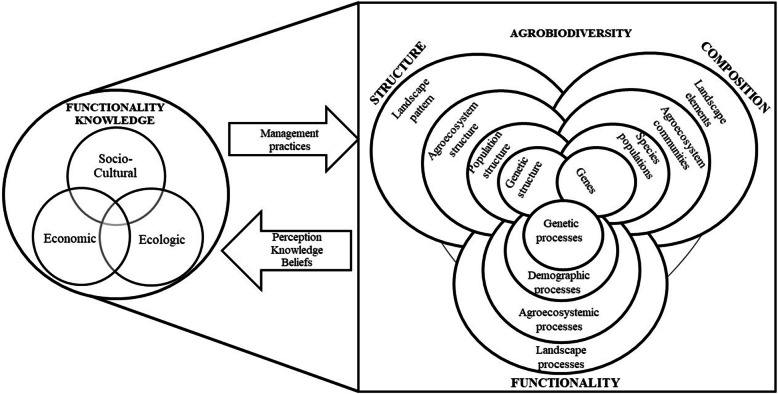
Relationship of HG agrobiodiversity and functionality knowledge of their owners (modified after Noss [18])

Agrobiodiversity and knowledge of its multifunctionalities are the result of continuing changes, whereby each acquires properties that modify both [25, 32, 33] (Fig. 1). People’s knowledge with regard to home gardens’ ecological, economic and socio-cultural functionality, i.e. knowledge of the system`s multifunctionality, evolves in a process of continuous transmittance and renewal in regional bioculture [24, 29, 34–37]. It responds dynamically to contextual influences on its production and reproduction, as described in literature on Traditional Ecological Knowledge (TEK) and Local Ecological Knowledge [38]. Berkes [38] considers that TEK includes associated practices and beliefs. Aldasoro-Maya [39] assigns the connotation of “contemporary” to these localized bodies of knowledge, emphasizing that although having roots in tradition, they also reflect interactions with other forms of knowledge, cultures and temporality, thus updating their suitability for the actual management of agrobiodiversity conservation. Multifunctionality knowledge is a part of these continuously actualized localized bodies of contemporary knowledge and contributes to the maintaining and renewing of biocultural diversity [24, 31, 40] and the diversification, updating and adaptation of socio-ecological systems such as home gardens [2, 41, 42].

In this article, we analyse how owners’ knowledge of HG multifunctionality functions links to HG agrobiodiversity, based on species inventory data from a sample of HGs in the tropical lowlands of México and the information provided by their owners on the ecological, economic and socio-cultural HG functions they distinguish and how they value them. In our hypotheses, HG multifunctionality knowledge is positively correlated with species richness and Shannon diversity index, as both enhance each other, and also relates with the species composition of HG flora as this reflects owners’ knowledge. Through our research, we pretend to contribute elements and methods for the design of policies and actions for agrobiodiversity conservation at the local level based on knowledge of HG multifunctionality as defined in their own terms by rural families from their livelihood strategies onwards [35, 43].

## Methods

### Study area

**Fig. 2:**
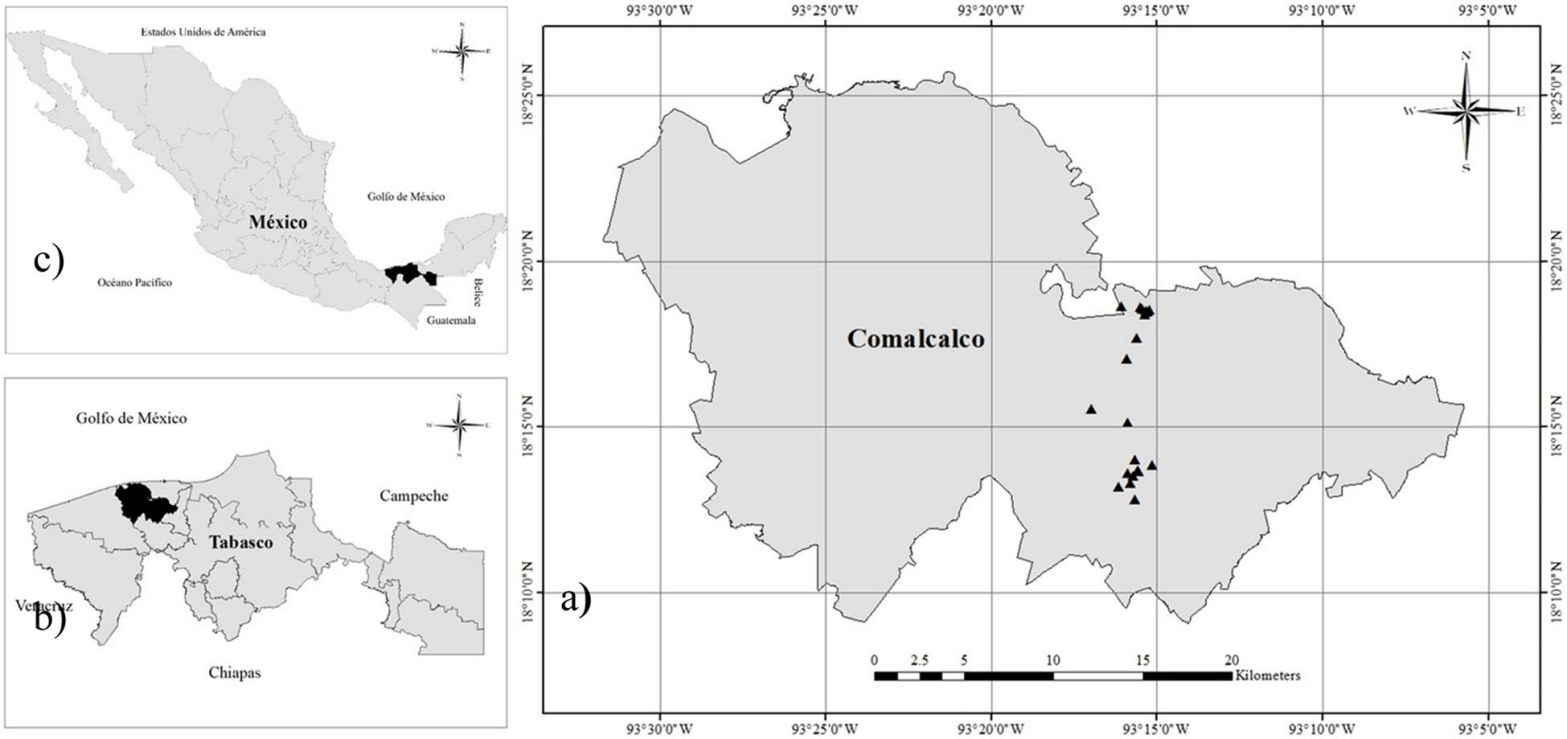
Study area. **a**) Location of studied HGs in Comalcalco. **b**) Location of Comalcalco in Tabasco. **c**) Location of Tabasco in México. Elaborated in ArcMap 10.1 [44].

We conducted fieldwork in the Comalcalco municipality in the heart of the cacao-producing tropical lowlands of the state of Tabasco, México. Based on the experience of the local NGO “Horizontes Creativos”, engaged in grassroots organization on social innovation, México, we selected the villages Zapotal, Gregorio Méndez, Reyes Hernández and Sargento López (Fig. 2), where research fitted in ongoing organization processes. The villages are located on the slightly elevated margins of former riverbeds, with fertile vertisols and gleysols [45]. The climate is hot and wet, with an average year temperature of 27.1 °C and annual rainfall of 1926 mm [46].

Agricultural modernization from the 1950s onwards has resulted in the general deforestation of the area originally covered with tropical rainforests [45, 47], home gardens and cacao plantations providing the remaining tree cover. Dominant land use is for animal husbandry and sugarcane, banana and cacao production. Villagers combine agricultural activities with work in the services sector and oil industries, Tabasco being Mexico’s main oil producer. The advent of the oil industry in the 1970s, its expansion and crisis have strongly impacted society, culture and environment. Deforestation, floods, contamination and lack of economic opportunities have recently catalysed social reactions such as the establishment of cooperatives and local micro-financing organizations.

### Home garden selection

We informed HG owners on the goals of the research at local meetings in January 2017 on responses to the productive crisis in cacao. We mentioned methods, research needs and possible benefits for local organization processes and invited the assistants to participate. Other families showed interest as information spread, reaching a total of 20 families: 8 in Zapotal, 3 in Gregorio Méndez, 4 in Reyes Hernández and 5 in Sargento López (Fig. 2). The total area of the 20 HGs was 5.2 hectares, considered sufficient to capture regional species richness to a large extent [48].

### Agrobiodiversity census

From February to June 2017, we registered all trees, shrubs, climbers, small woody shrubs (suffrutescents) and perennial herbs in each HG by local and scientific name, based on the knowledge of the research team, home garden owners and local experts. In case of doubt (occurring with less than 10 species), we took photographs and samples of leaves, flowers and/or fruits, which the first author compared with voucher specimens available in the herbarium at the Universidad Juárez Autónoma of Tabasco (UJAT). We checked the scientific names and biogeographical distribution of species (native, neotropical or introduced), at Trópicos [49], WCS [50] and The Plant List [51], and species’ conservation status considering the IUCN Red List [52], CITES [53] and NOM-059-SEMARNAT-2010 [54]. We registered intraspecific variation based on local cultivar names, colour and shape of leaves, flowers and fruits [2].

### Cataloguing home garden functions

We generated a draft catalogue of home garden functions from literature [7, 25, 26, 30, 32, 55–57], distinguishing ecological, economic and socio-cultural dimensions. From June to September 2018, we applied semi-structured interviews to the owners in their home garden to assure a connection, which we recorded with previous consent and transcribed. This allowed elaborating the final version of the catalogue, adding the functions that owners described and modifying or removing others. We interviewed household heads, in total 11 women and 19 men (see supplementary material “Agrobiodiversity data”, sheet HG), with ages ranging from 38 to 86 years. All interviewed families managed cacao groves; 9 also dedicated to other agricultural production systems and 11 to work in the industrial and services sectors.

Based on the transcribed interviews, we assigned a score to the knowledge of the functions in each dimension (socio-cultural, ecological, economic), following the general method of explicit assessment outlined by Bosshard (54). We assigned a score 0 if functions were not recognized, even negated; 1 if a function was not recognized, but the home garden reflected management regarding the function; 2 if a function was recognized, but the management did not reflect it; and 3 if owners recognized the function and the management reflected it. As for the economic dimension, we grouped 33 different uses of HG species in the economic functions of wood (5 uses), food (4), ornamentals (4), medicines (4), agricultural inputs (4), domestic products (4), handicrafts (4) and others (4) and based scores on the number of uses of species for each group mentioned by the home garden owners. We thus obtained aggregated scores for each dimension in all HGs, which we standardized to the same scale for the three dimensions, and then calculated an aggregated functionality knowledge score by summing the scores in the three dimensions.

### Data analysis

We elaborated a species abundance matrix from the inventory data and evaluated our sampling effort [58] by dividing the number of observed species by the estimated total regional home garden species richness using the Chao1 algorithm in the EstimateS program [59]. We used non-metric multidimensional scaling (NMDS) in the PAST software [60] on the species abundance matrix to represent variation in species composition among HGs along axes. We determined Spearman *rs* correlation coefficients between agrobiodiversity—considering observed species richness and Shannon H—and multifunctionality knowledge scores, using the PAST software. We also analysed if species composition was related to HG multifunctionality knowledge, by determining correlations of HG scores along the axis of NMDS with the knowledge scores for the ecological, economic and socio-cultural dimensions and their aggregate multifunctionality values.

### Knowledge dialogues at workshops

We shared the results of agrobiodiversity censuses and interviews with HG owners in workshops conducted in May and July 2018 and established a dialogue of different ways of knowing between HG owners and academics [61] on the relevance of relations of HG agrobiodiversity and multifunctionality knowledge for the management of the conservation of agrobiodiversity.

## Results

### HG agrobiodiversity

We registered 4349 individuals belonging to 280 botanical species, 229 genera and 84 families (Additional file 1 “Species list.xlsx”) [62]. Chao-1 total estimated species richness was 348, indicating that our sample included 80.7 % of all species in regional home gardens. Average species richness in HGs was 59.6 ± 5.1, of which 49 ± 4 were tree or shrub species (Additional file 2 “Agrobiodiversity data.xlsx”) [63]. The average number of perennial individuals in HG was 217.5 ± 23.8. HG size was on average 2584 m^2^ and showed no significant correlation with the number of individuals (*rs* = 0.14, *P* = 0.56) and total species richness (*rs* = − 0.08, *P* = 0.71). Families with smaller HGs compensated with higher numbers of individuals (*rs* = − 0.54, *P* = 0.01) and of species of perennial herbs (*rs* = − 0.46, *P* = 0.04). The average Shannon diversity index was 3.35, with a minimum of 2.30 and a maximum of 4.02.

Of the 280 inventoried species, 33.2% were native to Mesoamerica, 26.4% of neotropical origin and 40.4% introduced (Additional file Species list.xlsx). Most abundant species were cacao, *Theobroma cacao* L., and macuilis, *Tabebuia rosea* (Bertol.) Bertero ex A.DC., with 6.3% and 6.2% of the total number of individuals, respectively, followed by *Citrus* x *sinensis* (L.) Osbeck (4.4%), *Ixora coccinea* L. (3.5%), *Cedrela odorata* L. (3.4%), *Cocos nucifera* L. (3.2%), *Eugenia rubella* Lundell (3.1%), *Annona muricata* L. (2.9%), *Mangifera indica* L. (2.5%), *Hibiscus rosa-sinensis* L. (2.2%) and *Melicoccus oliviformis* Kunth (2.1%). Together, these 11 species represented 39.9% of all individuals. *Citrus* x *sinensis* and *Tabebuia rosea* were present in all HGs. *Annona muricata* and *Mangifera indica* were present in 19 HGs, *Citrus* x *aurantium* L. in 18 HGs, and *Cedrela odorata*, *Citrus reticulata* Blanco, *Melicoccus oliviformis* Kunth, *Psidium guajava* L. and *Spondias purpurea* L. in 17. Sixty-eight species were only present with one individual and 32 with two. Of all registered species, 21 had some national or international conservation status [52–54, 62].

Growth habit of 43% of all inventoried species was arboreal or arborescent: 22% were shrubs, 10% suffrutescents (small shrubs), 9% climbers and 15% perennial herbs (see Additional file “Species list.xlsx”) [62]. The average proportion of arboreal and arborescent species in home gardens was 63%, shrubs 19%, perennial herbs 8%, suffrutescents 5% and climbers 5%. There was a considerable intraspecific diversity: HG owners had local names for 65 cultivars of 14 species, with *Mangifera indica* accounting for 16.

### HG multifunctionality knowledge

We recorded a total of 38 HG functions (f), of which 14 were in the ecological, 12 in the economic and 12 in the socio-cultural dimension (Table 1). The scales for determining knowledge scores for the socio-cultural, economic and ecological functions are based on the deliberations in the research team, and the interviews and are detailed in the Additional file 3 “Functionality knowledge data.xlsx”, sheets “function scores” and “scores by use groups” [64].

**Table 1:** Home gardens’ functions recognized by their owners in Comalcalco, Tabasco, México

*Dimension*	Code	Description
*Socio-cultural*	f1	Provide space for ludic, artistic, reflexive, relaxing and sport activities
	f2	Contribute socio-cultural elements for food sovereignty
	f3	Beautify the direct family and community environment
	f4	Strengthen family/community cohesion through common activities
	f5	Generate positive emotions and feelings
	f6	Strengthen human relations through gifts and exchange of products
	f7	Equitably sharing tasks in HG maintenance
	f8	Maintain cultural heritage
	f9	Maintain ecological knowledge and wisdom
	f10	Ambience to transmit contemporary knowledge to new generations
	f11	Site for family and community traditions
	f12	Provide ingredients for culinary traditions
Ecologic	f13	Provide mineral nutrients from soil and water
	f14	Provide food and living space for wildlife
	f15	Conserve agrobiodiversity
	f16	Filter atmospheric contamination
	f17	Attract regional rainfall
	f18	Free oxygen, absorb carbon dioxide and produce biomass
	f19	Equilibrate provision of nutrients by providing organic matter
	f20	Maintain soils’ physical-chemical properties
	f21	Mitigate the impact of strong winds
	f22	Provide variation in microclimate
	f23	Receive and disperse seeds
	f24	Regulate physiological conditions of establishing, juvenile plants
	F25	Regulate temperature through transpiration and filtering radiation
	F26	Rehabilitate tree cover
Economic	F27	Provide products for selling to contribute to income
	F28	Contribute economically to food sovereignty
	F29	Facilitate the continuous availability of wood for local use
	F30	Provide a diversity of products through the year
	F31	Provide plants for use in agriculture (tools)
	F32	Provide plants for the production of handicrafts
	F33	Provide edible plants
	F34	Provide plants for domestic uses (utensils)
	F35	Provide wood
	F36	Provide medicinal plants
	F37	Provide ornamental plants
	F38	Provide plants for other uses

**Fig. 3:**
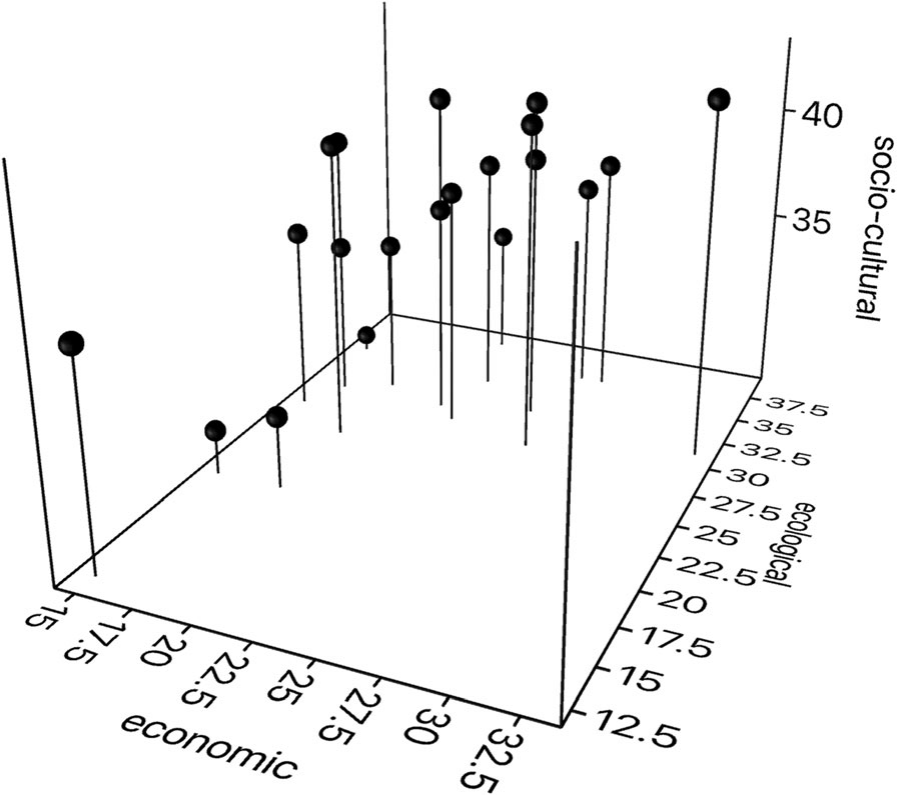
Functionality knowledge scores in the economic, ecological and socio-cultural dimensions among home garden owners in Tabasco, Mexico

The total HG functionality knowledge scores varied from 64.1 to 106.6, resulting from variable combinations of scores in the three dimensions (Fig. 3). Coefficient of variation was highest in the economic dimension (24.5%), followed by the ecological dimension (19.8%) and the socio-cultural dimension (11.7%). Though HG owners considered that socio-cultural, economic and ecological functions are all important at the same time, the medians of standardized absolute functionality knowledge scores in the three dimensions were different (Kruskal-Wallis test, *P* < 0.001; pairwise comparison with the Mann-Whitney test, *P* < 0.001 in all cases). The medians of economic, ecological and socio-cultural functionality knowledge scores were respectively 21, 31.1 and 36.9. Absolute scores of functionality knowledge in the economic dimension and in the ecological and socio-cultural dimension were positively correlated (*rs* = 0.44 and 0.64, respectively, *P* < 0.05), and there was no correlation between the ecological and socio-cultural knowledge scores (*rs* = − 0.03, *P* = 0.91).

In the socio-cultural dimension, all owners consider the maintenance of Traditional Ecological Knowledge and wisdom with regard to the environment as an important role of the HGs (f9, see Additional file 3 “functionality knowledge data.xlsx”, sheet “function scores”) [64]. All interviewees considered HGs’ impulse social learning among generations on agricultural themes and the taking care of productive spaces (f10), while several interviewees commented on the increasing difficulty to motivate young generations for these issues. HG activities enhanced family organization in 50% of the home gardens, where families distributed activities according to members’ capacities (f7). Most families consider that collective activities in home gardens strengthen family cohesion and organization (f4) and that gifts of HG products to family members, neighbours, friends and visitors strengthen human relations (f6) (see Additional file 3 “functionality knowledge data.xlsx”) [64].

HGs provide space for recreational, ludic, sports, artistic, religious, relaxing and social-familiar activities (meetings) in almost all HGs (f1). They host family and community traditions and customs (f11), such as the altar at Día de Muertos, religious services, Christmas, Easter and death wakes. At feasts in honour of the villages’ patron saints, products from home gardens are brought to the church as offerings or for sale to fund religious activities. Many owners considered HGs to represent a cultural and historical legacy of their ancestors (f8).

All HG owners recognized the aesthetic function, as HGs beautify the families’ direct environment and the village landscape (f3). HGs stimulate the senses and generate positive emotions and feelings that favour mental and spiritual health (f5). In this context, people mention terms such as wellbeing, peace, tranquillity, satisfaction, confidence, security, nostalgy, yearning, identity, pride, love, happiness, joy, concentration, inspiration, pleasure, harmony and company, among others. The emotional function (f5) received the highest allover score (60) of all functions and numerous expressions of HG owners during the interviews support them.

HGs’ contribution to food sovereignty involves the socio-cultural and economic dimensions, as mentioned by all families. In the socio-cultural dimension, families considered that the foods produced in HGs have cultural value and contribute to a diverse diet, influencing positively in health and satisfying the local sense of taste (f2). Another socio-cultural function is to contribute to maintain culinary traditions (f12), using cultivated ingredients as a part of the process that involves the traditional kitchen structures, organization and transmission of knowledge. In the economic dimension, HGs contribute to regional and local food autonomy reducing daily expenditures (f28).

One of the economic functions is providing income from sales of HG products (f27). Families mention that its contribution to income is small, yet important as it continues all over the year due to HG species richness (f30). Forest similarity of HGs, with trees of different ages and architecture, allows spreading the harvesting of wood in time according to needs (f29) or use the incrementing wood stock as a piggy. Wood supply has the highest score among the economic functions (f35) and is reported by all families to meet needs for carpentry (e.g. *Cedrela odorata* and *Swietenia macrophylla*), energy (e.g. *Diphysa americana*), construction (e.g. *Colubrina arborescens*) and fences (e.g. *Bursera simaruba* and *Gliricidia sepium*, *Nopalea cochenillifera*, *Sansevieria zeylanica*). Food provision had the second highest score (f33), and includes fruits (e.g. *Citrus* spp. and *Annona* spp.), leaves (e.g. *Piper auritum*), stems (e.g. *Saccharum officinarum*), roots (e.g. *Manihot esculenta*), condiments (e.g. *Plectranthus amboinicus* and *Pimenta dioica*), inputs for sweets (e.g. *Vasconcellea pubescens* and *Malpighia glabra*), ferments (e.g. *Theobroma cacao* and *Byrsonima crassifolia*) and wrapping materials (e.g. *Piper auritum*, *Calathea lutea* and *Musa* spp*.*).

All HGs provide ornamental plants (f37). The category includes ornamentals *sensu stricto* (e.g. *Hibiscus* spp., *Ixora* spp. and *Rosa* spp.), aromatic (e.g. *Cestrum nocturnum*) and ritual species (e.g. *Bursera graveolens* and *Cordyline fruticose*), as well as species that people maintain as a “relic”, i.e. plants maintained as a living memory, be it for their sentimental value towards the person who planted it, or because the plants have become increasingly rare so it is necessary to learn new generations about them (e.g *Aristolochia pentandra* and *Smilax domingensis*).

All owners mention the HGs’ provision of plants of medicinal uses (f36), be these of curative (e.g. *Tradescantia spathacea*, *Citrus* x *aurantium* and *Sambucus canadensis*), cosmetic (e.g. *Aloe vera*), relaxing (e.g. *Justicia pectoralis*) or energizing (e.g. *Theobroma cacao*). However, several interviewees manifested that the new generations lack knowledge about these plants as they stop using and cultivating them. The function of providing products for use in agricultural production (f31) includes plant parts as tools (e.g. *Genipa americana*), forage (e.g. *Gliricidia sepium*), green manure (e.g. *Erythrina caribaea*) and control of plagues (e.g. *Azadirachta indica*). Domestic uses (f34) include the use as insect repellents (e.g. *Ocimum basilicum*), utensils and containers (e.g. *Cocos nucifera*), basketry (e.g. *Sabal mexicana*) and fibre for ropes (e.g. *Heliocarpus appendiculatus*). Plants for handicrafts (f32) provide plant colorants (e.g. *Bixa orellana*), materials to elaborate handicrafts (e.g. *Crescentia cujete*), toys (e.g. *Canna indica*) and music instruments like traditional drums (e.g. *Persea americana*). The functions of provision of materials for domestic uses and handicrafts obtained the lowest scores (Additional file 3). Though interviewees mentioned the use of plants for these functions, they also observed that they are available in very few HGs and not in theirs. Rather, many materials traditionally used for these functions have been substituted by widely available and cheap plastic objects. As plants for other uses (f38), families mentioned shade trees (e.g. *Terminalia catappa*), melliferous plants (e.g. *Lonchocarpus hondurensis* and *Melicoccus* spp.), stinging (e.g. *Phenax hirtus*) and oil-providing plants (e.g. *Cocos nucifera* and *Acrocomia aculeata*).

Temperature regulation scored highest among the ecological functions (f25). Interviewees commented how tree cover filters the sunlight and avoids heating of the soils. Transpiration by plants is considered to lower the air temperature, as well as interception and introduction of air flows by trees, replacing hot air and mitigating high temperatures. Well-positioned trees protect houses and other structures from strong winds (f21). Owners also consider that HGs filter atmospheric contamination (f16), as leaves absorb and trap suspended particles. Owners also mentioned the production of oxygen and the absorption of carbon dioxide as important functions (f18) and observed that HGs attract rainfall (f17) as does other forest-like vegetation. In their view, deforestation has shortened rainy seasons, lengthened dry periods and has caused higher temperatures, affecting crop production and human health.

Families consider that HGs conserve agrobiodiversity (f15), as they maintain plants that do not occur any more in other spaces of the regional landscape. HGs are a source of seeds that colonize adjacent fields and receive seeds from other fields (f23). They contribute to conservation of associated agrobiodiversity by providing food and refuge for fauna (f14), including birds, mammals, reptiles, monkeys, native bees and other species that are tolerated or favoured. Families also mentioned that they routinely eliminate species that cause damage to the vegetation or domestic animals, or that are dangerous for humans, mentioning snakes, squirrels, rats, possums and rapacious birds.

HGs’ vegetation structure with big and small trees in dense and open patches generates variation in micro-climatic functions (f22), and thus the provision of adequate conditions for species with different physiological requirements (f24). For example, *Citrus* spp. require direct sunlight for optimum fruit production, while cacao (*Theobroma cacao*) needs a certain degree of shade. Open spaces are often used for ornamentals and also for productive activities such as the drying of fresh cacao beans.

HGs thrive on soils that widely vary in texture and fertility (f20). Families have broad knowledge of this variation, which orients the selection of sites for planting particular species and of management practices to adapt to limitations. For example, owners may maintain a cover crop on soils with a fine sandy texture to avoid high temperatures of such soils, conditions to which e.g. *Citrus* spp. and *Pimenta dioica* are not adapted. Soils function as the main reservoir of nutrients and water for plants (f13), and families also consider that rains, floods and air play a role in the provision of both. HG vegetation contributes to soil fertility by providing organic matter (f19), thus contributing to maintain soil humidity and porosity and avoid erosion. Though families recognize this function, the removal and burning of leaf litter is a general practice, and only few families return decomposed organic materials to the plants. The main reason for burning is to avoid the spread of snakes and mosquitos. Owners consider that HGs act as vehicles to rehabilitate or maintain tree cover (f26). Their establishment frequently involves the replacement of some of the naturally occurring trees with trees of more useful species but may also start from tree planting on formerly deforested areas.

### Relations of HG agrobiodiversity and multifunctionality knowledge

Within each dimension, we standardized absolute scores to a potential maximum score of 45 (Additional file “Functionality knowledge data.xlsx”, sheet “function scores by dimension” [64] and tested these scores for relations with species diversity and composition.

In general, HG owners perceive that the more species of plants, the more functions are met that benefit the people and the environment. Correlation analysis on the data of all home gardens showed indeed significant correlations of richness and Shannon diversity indexes with knowledge scores in particular dimensions (Table 2). Species richness and Shannon H were significantly correlated with ecological functionality knowledge (*rs* = 0.68 and 0.68, *P* < 0.001); species richness was also correlated with economic functionality knowledge (*rs* = 0.47, *P* = 0.03), but not with the socio-cultural and the aggregated functionality knowledge scores (*rs* = − 0.18 and 0.44, *P* = 0.45 and 0.052). Shannon H was also not correlated with socio-cultural functionality knowledge (*rs* = − 0.24, *P* = 0.31). When separating species richness by the biogeographical origin of the species (see Additional file 1, Species list), the observed correlation pattern was maintained, i.e. independently of the origin, there were no correlations of richness with socio-cultural functionality and significant correlations with ecological functionality (Table 2). Additionally, there were significant correlations between aggregated functionality and richness of native species and of the sum of native and neotropical species richness.

**Table 2:** Spearman *rs* correlation coefficients of home garden species richness and Shannon diversity indexes with functionality knowledge scores of their owners in Comalcalco, Tabasco

*Variables*	*Socio-cultural functionality*		*Ecological functionality*		*Economic functionality*		*Multifunctionality*	
	*r*_*s*_	*P*	*r*_*s*_	*P*	*r*_*s*_	*P*	*r*_*s*_	*P*
Observed richness	− 0.18	0.45	0.68	0.001	0.47	0.03	0.44	0.052
Shannon H	− 0.24	0.31	0.68	0.0002	0.40	0.08	0.39	0.09
Native	− 0.07	0.77	0.73	0.0003	0.56	0.01	0.54	0.01
Neotropical	− 0.41	0.07	0.66	0.002	0.17	0.47	0.18	0.46
Native-neotropical	− 0.12	0.60	0.74	0.0002	0.49	0.03	0.48	0.03
Introduced	− 0.16	0.50	0.57	0.01	0.46	0.04	0.40	0.08

NMDS, applying the Bray-Curtis similarity index and three dimensions, gave a stress factor of 0.21, indicating a reasonable representation of variation in species composition. Species composition scores on the first and second axes and ecological functionality knowledge showed significant correlations, with *rs* = 0.49 and *P* = 0.03 on the first axis and *rs* = − 0.49, *P* = 0.03 on the second axis. Other functionality knowledge scores showed no correlation with NMDS scores.

Exchange of knowledge on functionality and agrobiodiversity relations in workshops of HG owners, the research team and NGOs allowed discussions on how to assemble ideal HGs. This exercise showed the desirability of enhancing and combining many of the functions mentioned in the function catalogue (Table 1). Owners emphasized the importance of knowledge on the production of wood and fruit in the economic dimension, the provision of living space for native stingless bees and other biological groups in the ecological dimension, and on how to transmit knowledge from generation to generation in the socio-cultural dimension. Together, these components of functionality knowledge enhance agrobiodiversity conservation in HG, thus allowing the knowledge to be maintained and evolve.

## Discussion

Agrobiodiversity in our sample of 20 HGs in Tabasco was quite high (279 species) as compared to the findings in other studies in México [65, 66] and the tropics in general [2, 3, 11, 24, 37, 67]. The richness of species native to Mesoamerica and the Neotropics and the fact that 21 species are listed in national and international conservation categories confirm HGs’ high relevance for regional conservation of agrobiodiversity as well as ongoing species domestication [2, 25, 31]. The many uses of plant species distinguished by the home garden owners (33, Additional file 3) and the presence of many cultivars of HG species that are adapted to the regional environmental conditions reflect how the knowledge on functions of HGs is very much alive.

HGs maintain the regional agrobiodiversity that people consider important [2–4, 24, 68]. Based on functionality knowledge, owners select the species and cultivars for their HGs [25, 35]. In the study area, this notably includes species of different growth habits: 33% of the inventoried species were suffrutescents, climbers or perennial herbs. The combination of different growth habits allows owners to adjust species selection to the available area and explains why even small HGs are rich in species. Families with smaller HGs compensated with higher numbers of individuals and species of perennial herbs, explaining why HG size showed no significant correlation with the number of plant individuals nor total species richness. Due consideration of different growth forms in HG agrobiodiversity studies therefore allows a more complete view of their management [3].

Species abundance was highly skewed: 39.9% of the inventoried plants belonged to only 11 species and were present in most HGs, whereas 100 species were singletons or doubletons. Singletons and doubletons were not equally distributed over home gardens: three home gardens had together 36 singletons and concentrated more than a third of doubletons. Only a few HGs had no singleton (1) or doubleton (3). This shows that HG owners intentionally care for rare species and some dedicate special effort on this task, based on knowledge of their contribution to HG multifunctionality. Examples of rare species taken care of are *Acrocomia aculeata*, *Annona purpurea*, *Aristolochia pentandra*, *Chrysophyllum mexicanum*, *Dioscorea composita*, *Garcinia intermedia* and *Smilax domingensis*, among others.

In this article, we have used the term “multifunctionality knowledge” in the three distinguished dimensions (ecological, economic, socio-cultural), which add up to multifunctionality knowledge. As mentioned in the introduction, this knowledge is part of what has been referred to in literature as Traditional Ecological Knowledge (TEK) or Local Ecological Knowledge. Berkes [38] considers them as complex bodies of knowledge, belief and practices that is culturally transmitted and warns to consider them as static. Rather, TEK (or LEK) is frequently reinvented and adapted to meet changing needs and is in this sense contemporary knowledge. Far from being static, it is reproduced, enriched and renewed continuously, integrating new elements [35, 38, 40]. Based on our results, we would prefer a term for TEK/LEK that reflects the integration of economic, ecological and socio-cultural aspects and the fusion of traditional, contemporary and scientific elements in the localized knowledge body, as they all influence natural resource management. We would avoid the exclusive epithet “ecological”, as it narrows the integrality of local systemic knowledge.

Multifunctionality knowledge guides practices and is transmitted through narratives, observations and learnings that are part of and renew a social memory [35]. HG owners had knowledge on 38 functions in the ecological, economic and socio-cultural dimensions (Additional file 3). In their vision, all are part of the same integral knowledge system, and for this reason, some of the functions are transversal to the dimensions or play a role at different scales (Fig. 1), including the cultural landscape.

We refer in results that families have a strong consideration of socio-cultural functions as compared to ecological and economic functions. This confirms findings in the Catalan Pyrenees, where cultural aspects were also most valued [56]. A difference was that the Pyrenees owners did not consider climate regulation and provision of habitat as relevant, as HGs were small—on average 147 m^2^—as compared to the surrounding forested areas. This contrasts strongly with the Tabasco context, where man-made forests like HGs and cacao groves provide most forest cover [45, 47] and comply with the ecological functions formerly provided by the natural vegetation. This shows how functionality knowledge in the three dimensions reflects the regional context. Functionality knowledge and agrobiodiversity are dialectically related: due to deforestation and broader societal changes, owners start to consider new functions as important and procure them in their HGs, which thus acquire new characteristics, and may eventually influence the more general context. For example, it is notorious how owners presently consider seed rains from home gardens to their species-poor surroundings as an ecological function (Additional file 3).

Overall change in the socio-ecological context in Tabasco in recent decades (deforestation, oil industries, contamination, migration, urban-rural relations) has influenced the renewal of functionality knowledge and agrobiodiversity in HGs and, broader, in the cultural landscape. Updating of functionality knowledge makes use of the available sources in daily social interactions. One of these is the transmittance of knowledge by elders to the new generations. HG owners referred frequently to this in wordings as “our ancestors said”, “gone generations knew”, “the old tell us” and “our parents taught us”. Another source is the knowledge of professionals transmitted through environmental and agricultural projects, referred to as “as the engineer says”, “in the course they taught us” and “according to the technical officer”. Another source is the interaction with academics and NGOs working on socio-ecological themes, local organizations that have gone through processes of adjustments of practice and knowledge, through meetings and cultural events and internet access. Also, children and young adults transmit new knowledge, for example with regards to gardening with recycled materials. These instances of dialogues of different ways of knowing [61] and access to information allow the updating of multifunctionality knowledge applied to and interacting with HG agrobiodiversity.

The high score of functionality knowledge in the socio-cultural dimension and in particular of knowledge transmission to younger generations indicate that HGs are a response to the ecological, economic and social changes in Tabasco [47], as has occurred also in other regions [12, 67, 69]. This response explains why we did not find a correlation between species richness/Shannon diversity index and functionality knowledge scores in the socio-cultural dimension: much of the considered functionality knowledge does not depend directly on the availability of species in the HG but rather refers to the belonging to a regional culture and the desire to transmit it (for example, f1, f3, f4, f5, f7 and f11). Socio-cultural functionality knowledge is thus a strong asset for the strengthening of regional bioculture among the new generations.

Economic functionality knowledge scores were low as compared to those for socio-cultural and ecological functionality knowledge (Fig. 3). The relatively high variation coefficient indicates that some HG scarcely comply with economic functions and others considerably (Fig. 3). This variation may be partly due to the small contributions of HGs to family economy in small HGs, as indicated by a significant correlation of economic functionality knowledge scores and HG area (*rs* = 0.510, *P* = 0.026). Economic functionality knowledge was also significantly correlated with species richness. Scores were highest for wood and fruit provision functions and lowest for handicrafts and domestic uses, as substitutes of the latter are readily available in local shops [5, 17, 25]. HGs’ contribution to family economy through sales or savings in spending thus contributes to agrobiodiversity, but this may combine with the substitution of species.

The relations of agrobiodiversity and functionality knowledge show many variations among families, as these participate differently of regional bioculture and knowledge of the socio-ecological system elements. In general, HG species richness and owners’ total functionality knowledge scores showed no significant correlation (*rs* = 0.44, *P* = 0.052) (Table 2). We had expected the contrary: owners managing more species would consider more functions, resulting in higher total functionality knowledge scores. The absence of such an overall correlation is due to several factors. As mentioned earlier, part of functionality knowledge goes beyond the individual home garden (Additional file 3) and is not necessarily related to species richness, as in the case of several socio-cultural functionalities. Also, the presence of multi-purpose species [33] explains that higher functionality knowledge scores are not necessarily associated with higher species richness, as does the situation where owners maintain trees without having knowledge on their functionality [17, 32, 70].

Scores of the economic and ecological functionality knowledge showed significant correlations with species richness, and in the ecological dimension also with the Shannon H diversity index (Table 2). These correlations were also found when we separated species by their biogeographical origins, indicating that families experiment in their HGs with both regionally occurring and introduced species [71]. It is noteworthy that also aggregated functionality knowledge was significantly correlated with the richness of native species, reflecting the importance of this agrobiodiversity component in regional culture. Socio-cultural functionality knowledge scores were however not correlated with species richness. As observed above, these scores correlated positively with home garden area (*rs* = 0.71, *P* = 0.001), as was also the case of economic (*rs* = 0.522, *P* = 0.022) and total functionality knowledge scores (*rs* = 0.507, *P* = 0.027). Larger available area logically allows addressing more functions, often in specific home garden sections [72]. Several socio-cultural functions, such as receiving family, friends and neighbours (f4), and economic functions require specific areas.

The wide array of HG functions in the ecological, economic and socio-cultural dimensions that people distinguish reflects the importance of HGs in local livelihoods and the daily practices aimed at maintaining and adapting of their socio-ecological systems. As such, multifunctionality knowledge provides concrete opportunities for agrobiodiversity conservation in the local and regional spheres. Multifunctionality knowledge-agrobiodiversity relations are a point of departure for working towards integral strategies of agrobiodiversity conservation and improved livelihoods [8, 10, 31, 35, 41, 73]. Families in the study area are aware of this and therefore actively promote biocultural attachment among the new generations, dedicating time to conserving, learning and teaching about HG functionality and establishing alliances with NGOs, academics, consumers and institutions to do so. Examples in Tabasco include initiatives that involve new generations in agricultural and conservation activities, such as the “School of peasant life” established in one of the study communities, as well as co-organized agroecology research and workshops. Sharing and advancing multifunctionality knowledge regarding HGs, as well as other socio-ecological systems, and their agrobiodiversity, is therefore a starting point and a central element for improving and adapting local livelihoods [24, 36, 73].

## Conclusions

The rich agrobiodiversity of home gardens cherished by rural families in Tabasco is positively correlated with HG owners’ broad multifunctionality knowledge in the ecological and economic dimensions. Although the socio-cultural functionality knowledge is not correlated with agrobiodiversity, its high scores underline the strong and general interest of local people in these aspects. The contemporary knowledge with regards to HG multifunctionality is a strong asset for the conservation of agrobiodiversity (43), as it is an integral part of local livelihoods. Its analysis should therefore be a starting point for policies and actions in this respect.

## Supplementary information

**Additional file 1.** List of the perennial species found in the sample of 20 home gardens in Comalcalco, Tabasco (file name: Species list.xlsx)

**Additional file 2.** Species and their abundances in the sampled home gardens (file name: Agrobiodiversity data.xlsx)

**Additional file 3.** Functionality knowledge scores of the owners of the sampled of home gardens (file name: Functionality knowledge data.xlsx)

## Data Availability

All data generated or analysed during this study are included in this published article and additional data files.

## References

[1] Casas A, Parra F, Torres-García I, Rangel-Landa S, Zarazúa M, Torres-Guevara J. Estudios y patrones continentales de domesticación y manejo de recursos genéticos: perspectivas. In: Casas A, Torres-Guevara J, Parra F, editors. Domesticación en el continente americano. Domesticación en el Continente Americano. Vol. 2. Ciudad de México, Lima: Universidad Nacional Autónoma de México, Universidad Nacional Agraria La Molina; 2017. p. 537-70.

[2] Watson JW, Eyzaguirre PB (2002). Home gardens and in situ conservation of plant genetic resources in farming systems.

[3] Kumar BM, Nair PKR (2004). The enigma of tropical homegardens. Agroforestry Systems..

[4] Montagnini F, Nair PKR (2006). Homegardens of Mesoamerica: biodiversity, food security, and nutrient management. Kumar B.

[5] Trinh LN, Watson JW, Hue NN, De NN, Minh NV, Chu P (2003). Agrobiodiversity conservation and development in Vietnamese home gardens. Agriculture, Ecosystems & Environment..

[6] Alcudia-Aguilar A, van der Wal H, Suárez-Sánchez J, Martínez-Zurimendi P, Castillo-Uzcanga MM (2018). Home garden agrobiodiversity in cultural landscapes in the tropical lowlands of Tabasco. México. Agroforestry Systems..

[7] Ordonez JC, Luedeling E, Kindt R, Tata HL, Harja D, Jamnadass R (2014). Constraints and opportunities for tree diversity management along the forest transition curve to achieve multifunctional agriculture. Current Opinion in Environmental Sustainability..

[8] Zimmerer KS. Conserving agrobiodiversity amid global change, migration, and nontraditional livelihood networks: the dynamic uses of cultural landscape knowledge. Ecology and Society. 2014;19(2).

[9] Sauer CO (1925). The morphology of landscape.

[10] Boege E. El patrimonio biocultural de los pueblos indígenas de México. Hacia la conservación in situ de la biodiversidad y agrodiversidad en los territorios indígenas: Instituto Nacional de Antropología e Historia - Comisión Nacional para el Desarrollo de los Pueblos Indígenas; 2008.

[11] Lamont SR, Eshbaugh WH, Greenberg AM (1999). Species composition, diversity, and use of homegardens among three Amazonian villages. Economic Botany..

[12] Moreno-Black G, Somnasang P, Thamathawan S (1996). Cultivating continuity and creating change: women’s home garden practices in northeastern Thailand. Agriculture and Human Values..

[13] Gbedomon RC, Salako VK, Fandohan AB, Idohou AFR, Glèlè Kakaï R, Assogbadjo AE. Functional diversity of home gardens and their agrobiodiversity conservation benefits in Benin, West Africa. Journal of Ethnobiology and Ethnomedicine. 2017;13(1). 10.1186/s13002-017-0192-5 PMC5702203 29178909

[14] Bidartondo MI, Read DJ, Trappe JM, Merckx V, Ligrone R, Duckett JG (2011). The dawn of symbiosis between plants and fungi. Biology Letters..

[15] Casas A, Parra F, Rangel-Landa S, Blancas J, Valejo M, Moreno- Calles AI, et al. Manejo y domesticación de plantas en Mesoamérica. Una estrategia de investigación y estado del conocimiento sobre los recursos genéticos. In: Casas A, Torres-Guevara J, Parra F, editors. Perspectivas de investigación y manejo sustentable de recursos genéticos en el Nuevo Mundo. Domesticación en el Continente Americano. vol 2. Morelia, Michoacán, México: Universidad Nacional Autónoma de México/Universidad Nacional Agraria La Molina/CONACYT; 2017. p. 69-102.

[16] Moreno-Calles AI, Casas A, Rivero-Romero AD, Romero-Bautista YA, Rangel-Landa S, Fisher-Ortíz RA, et al. Ethnoagroforestry: integration of biocultural diversity for food sovereignty in Mexico. Journal of Ethnobiology and Ethnomedicine. 2016;12(1). 10.1186/s13002-016-0127-6 PMC5120568 27881142

[17] Vandermeer J, van Noordwijk M, Anderson J, Ong C, Perfecto I (1998). Global change and multi-species agroecosystems: concepts and issues. Agriculture, Ecosystems & Environment..

[18] Noss R (1990). Indicators for monitoring biodiversity: a hierarchical approach. Conservation Biology..

[19] Goldstein PZ (1999). Functional ecosystems and biodiversity buzzwords. Conservation Biology..

[20] Thompson KM, Culley TM, Zumberger AM, Lentz DL. Genetic variation and structure in the neotropical tree, *Manilkara zapota* (L) P. Royen (Sapotaceae) used by the ancient Maya. Tree Genetics & Genomes. 2015;11:40.

[21] Rooduijn B, Bongers F, van der Wal H (2018). Wild native trees in tropical homegardens of Southeast Mexico: fostered by fragmentation, mediated by management. Agriculture, Ecosystems & Environment..

[22] Ortíz-Sánchez A, Monroy-Ortiz C, Romero-Manzanarez A, Luna-Cavazos M, Castillo-España P (2015). Multipurpose function of home gardens in the family subsistence. Botanical Sciences..

[23] Comberti C, Thornton TF (2015). Wyllie de Echeverria V, Patterson T. Ecosystem services or services to ecosystems? Valuing cultivation and reciprocal relationships between humans and ecosystems. Global Environmental Change..

[24] Galluzzi G, Eyzaguirre PB, Negri V (2010). Home gardens: neglected hotspots of agro-biodiversity and cultural diversity. Biodiversity Conservation..

[25] Huai H, Hamilton A (2009). Characteristics and functions of traditional homegardens: a review. Frontiers of Biology in China..

[26] Mander Ü, Wiggering H, Helming K (2007). Multifunctional land use. meeting future demands for landscape goods and services.

[27] Barberi P (2013). Functional agrobiodiversity: the key to sustainability? Agricultural Sustainability.

[28] Jax K (2005). Function and “functioning” in ecology: what does it mean?. Oikos..

[29] Tscharntke T, Clough Y, Bhagwat SA, Buchori D, Faust H, Hertel D, et al. Multifunctional shade-tree management in tropical agroforestry landscapes—a review. Journal of Applied Ecology. 2011;48(3):619-629.

[30] Brandt J, Vejre H (2004). Multifunctional landscapes. Theory, values and history.

[31] Casas A, Parra F, Blancas J. Evolution of humans and by humans. In: Albuquerque U, Paulino, Muniz de Medeiros P, Casas A, editors. Evolutionary etnobiology. Switzerland: Springer; 2015. p. 21-36.

[32] Hooper DU, Chapin FS, Ewel JJ, Hector A, Inchausti P, Lavorel S (2005). Effects of biodiversity on ecosystem functioning: a consensus of current knowledge. Ecological Monographs..

[33] Lamont BB (1995). Testing the effect of ecosystem composition/structure on its functioning. Oikos..

[34] Maffi L (2005). Linguistic, cultural, and biological diversity. Annual Review of Anthropology..

[35] Pretty J, Adams B, Berkes F, Athayde S, Dudley N, Hunn E (2009). The intersections of biological diversity and cultural diversity: towards integration. Conservation and Society.

[36] Moreno-Calles AI, Toledo VM, Casas A. Los sistemas agroforestales tradicionales de México: una aproximación biocultural. Botanical Sciences. 2013;91(4).

[37] Serrano-Ysunza AA, van der Wal H, Gallardo-Cruz JA, Ramos-Muñoz DE, Vaca RA (2018). A 6-year longitudinal study on agrobiodiversity change in homegardens in Tabasco. México. Agroforestry Systems..

[38] Berkes F (2008). Sacred ecology 2nd edition ed.

[39] Aldasoro-Maya EM (2012). Documenting and contextualizing Pjiekakjoo (Tlahuica) knowledges though a collaborative research project.

[40] Calvet-Mir L, Riu-Bosoms C, González-Puente M, Ruiz-Mallén I, Reyes-García V, Molina JL (2015). The transmission of home garden knowledge: safeguarding biocultural diversity and enhancing social–ecological resilience. Society & Natural Resources..

[41] van Noordwijk M, Hoang M, Neufeldt H, Öborn I, Yatich T. How trees and people can co- adapt to climate change: reducing vulnerability through multifunctional agroforestry landscapes. Nairobi, Kenya: World Agroforestry Centre (ICRAF); 2011. 152 p.

[42] Plieninger T, van der Horst D, Schleyer C, Bieling C. Sustaining ecosystem services in cultural landscapes. Ecology and Society. 2014;19(2).

[43] Díaz S, Demissew S, Carabias J, Joly C, Lonsdale M, Ash N, et al. The IPBES conceptual framework—connecting nature and people. Current Opinion in Environmental Sustainability. 2015;14:1-16.

[44] ESRI (2011). ArcGIS Desktop. Release 10 ed.

[45] Palma-López DJ, Cisneros-Domínguez J, Moreno-Cáliz E, Rincón-Ramírez JA (2007). Suelos de Tabasco: su uso y manejo sustentable.

[46] INEGI. Anuario estadístico y geográfico de Tabasco 2016. Aguascalientes, México: Instituto Nacional de Estadística y Geografía; 2016. 461 p.

[47] Tudela, F. La modernización forzada del trópico: el caso de Tabasco. Proyecto integrado del Golfo. 1992 ed. El Colegio de México: México; 1989. 478 p.

[48] van der Wal H, Bongers F (2013). Biosocial and bionumerical diversity of variously sized home gardens in Tabasco. Mexico. Agroforestry Systems..

[49] Tropicos [Internet]. Missouri Botanical Garden. 2015 [cited sept 2019]. Available from: http://www.tropicos.org.

[50] World Checklist of Selected Plant Families [Internet]. Royal Botanic Gardens. 2017 [cited september 2019]. Available from: http://wcsp.science.kew.org/.

[51] The plant list. A working list of all plant species [Internet]. The Plant List. 2013 [cited september 2019]. Available from: http://www.theplantlist.org/.

[52] The IUCN red list of threatened species. Version 2019-1 [Internet]. IUCN. 2019 [cited september 2019]. Available from: http://www.iucnredlist.org.

[53] Checklist of CITES species [Internet]. CITES-UNEP-WCMC. 2014 [cited september 2019]. Available from: http://checklist.cites.org/.

[54] Anomymous. Norma oficial mexicana NOM-059-SEMARNAT-2001 Protección ambiental—especies nativas de México de flora y fauna silvestres—categorías de riesgo y especificaciones para su inclusión, exclusión o cambio—lista de especies en riesgo. Diario Oficial de la Federación. 2002 6 march 2002.

[55] Bosshard A (2000). A methodology and terminology of sustainability assessment and its perspectives for rural planning. Agriculture, Ecosystems & Environment..

[56] Calvet-Mir L, Gómez-Baggethun E, Reyes-García V. Beyond food production: ecosystem services provided by home gardens. A case study in Vall Fosca, Catalan Pyrenees, Northeastern Spain. Ecological Economics. 2012;74(0):153-160.

[57] Carvalho-Ribeiro SM, Lovett A, O’Riordan T (2010). Multifunctional forest management in Northern Portugal: moving from scenarios to governance for sustainable development. Land Use Policy..

[58] Longino JT, Coddington J, Colwell RK (2002). The ant fauna of a tropical rain forest: estimating species richness three different ways. Ecology..

[59] Colwell RK. EstimateS: statistical estimation of species richness and shared species from samples. 9.1.0. ed. Colorado, USA2019.

[60] Hammer Ø, Harper DAT, Ryan PD (2001). PAST: Paleontological statistics software package for education and data analysis. Palaeontologia Electronica..

[61] Martínez-Torres ME, Rosset P (2014). Diálogo de Saberes in La Vía Campesina: food sovereignty and agroecology. The Journal of Peasant Studies..

[62] Avilez-López T (2019). Species list.

[63] Avilez-López T (2019). Agrobiodiversity data.

[64] Avilez-López T (2019). Functionality data.

[65] Castañeda-Navarrete J, Lope-Alzina DG, Díaz-Ordoñez MdJ. Los huertos familiares en la península de Yucatán. Atlas Biocultural de Huertos Familiares en México. 1. Cuernavaca: Universidad nacional Autónoma de México - CRIM; 2018. p. 331 - 390.

[66] Poot-Pool WS, van der Wal H, Flores-Guido S, Pat-Fernandez JM, Esparza-Olguin L (2015). Home garden agrobiodiversity differentiates along a rural-peri-urban gradient in Campeche. Mexico. Economic Botany..

[67] Panyadee P, Balslev H, Wangpakapattanawong P, Inta A (2018). Karen Homegardens: Characteristics, functions, and species diversity. Economic Botany..

[68] Trinh LN, Watson JW, Huec NN, Ded NN, Minhe NV, Chuf P (2003). Agrobiodiversity conservation and development in Vietnamese home gardens. Agriculture, Ecosystems and Environment..

[69] Kujawska M, Zamudio F, Montti L, Piriz CV (2018). Effects of landscape structure on medicinal plant richness in home gardens: evidence for the environmental scarcity compensation hypothesis. Economic Botany..

[70] Swift MJ, Izac A-M, Van Noordwijk M. Biodiversity and ecosystem services in agricultural landscapes—are we asking the right questions? Agriculture, Ecosystems & Environment. 2004;104(1):113-134.

[71] Albuquerque UP, Andrade LHC, Caballero J (2005). Structure and floristics of homegardens in Northeastern Brazil. Journal of Arid Environments..

[72] Méndez VE, Lok R, Somarriba E (2001). Interdisciplinary analysis of homegardens in Nicaragua: micro-zonation, plant use and socioeconomic importance. Agroforestry Systems..

[73] Zimmerer KS, de Haan S, Jones AD, Creed-Kanashiro H, Tello M, Carrasco M (2019). The Biodiversity of Food and Agriculture (Agrobiodiversity) in the Anthropocene: research advances and a conceptual framework. Anthropocene..

